# Green approach for synthesis of bioactive Hantzsch 1,4-dihydropyridine derivatives based on thiophene moiety via multicomponent reaction

**DOI:** 10.1098/rsos.170006

**Published:** 2017-06-14

**Authors:** M. G. Sharma, D. P. Rajani, H. M. Patel

**Affiliations:** 1 Department of Chemistry, Sardar Patel University, University Campus, Vallabh Vidyanagr, 388 120 Gujarat, India; 2 Microcare laboratory, Surat, Gujarat, India

**Keywords:** Green approach, Hantzsch reaction, one-pot multicomponent reaction, ceric ammonium nitrate, single crystal study, microbial study

## Abstract

A novel green and efficient one-pot multicomponent reaction of dihydropyridine derivatives was reported as having good to excellent yield. In the presence of the catalyst ceric ammonium nitrate (CAN), different 1,3-diones and same starting materials as 5-bromothiophene-2-carboxaldehyde and ammonium acetate were used at room temperature under solvent-free condition for the Hantzsch pyridine synthesis within a short period of time. All compounds were evaluated for their *in vitro* antibacterial and antifungal activity and, interestingly, we found that **5(b–f)** show excellent activity compared with Ampicillin, whereas only the **5e** compound shows excellent antifungal activity against *Candida albicans* compared with griseofulvin. The cytotoxicity of all compounds has been assessed against breast tumour cell lines (BT-549), but no activity was found. The X-ray structure of one such compound, **5a**, viewed as a colourless block crystal, corresponded accurately to a primitive monoclinic cell.

## 1. Introduction

More than a hundred years ago, the reaction to produce 1,4-dihydropyridines (1,4-DHPs) was reported by Arthur Hantzsch [1]. These are important precursors due to their pharmacological and biological activities, such as antihypertensive, anti-anginal and as calcium channel blockade for cardiovascular disease. Consequently, several clinically important drugs appeared on the market with variable new active functional groups in their main skeleton, such as Nicardipine, Nifedipine, Nimodipine, Felodipine, Isradipine and Amlodipine [2–5]. Recently, various attempts have been taken up to improve the Hantzsch reaction using different alternative processes [6–10]. However, most of these reactions were reported with new trends as one-pot multicomponent reactions (MCRs) in the last 10–15 years; these reactions were carried out with certain disadvantages like: longer reaction time, expensive catalyst, higher temperature and tedious workup procedure. Great diversity in MCRs [11–13], developed using various ionic liquids as various Lowry-Bronsted acids, with several advantages has been well documented [14–16]. The clinical significance of pyran, thiophene derivatives of 3-acetylcoumarine based on 1,4-DHPs was the concern that they were reported to have an equivalent cytotoxic effect as the standard CHS 828 against a breast cancer cell line [17].

The above synthetic applications and their medicinal importance encourage us to develop and strive for the enhancement of the pharmacologically bioactive novel 1,4-DHP compounds using MCR. Moreover, the thrust of our work was to enhance the reactivity with suitable electron-releasing and withdrawing groups in reactants to minimize the by-products, and diminish the time of reaction and use of solvent, to establish our protocol with more economy and with a green concept.

**Scheme 1.**
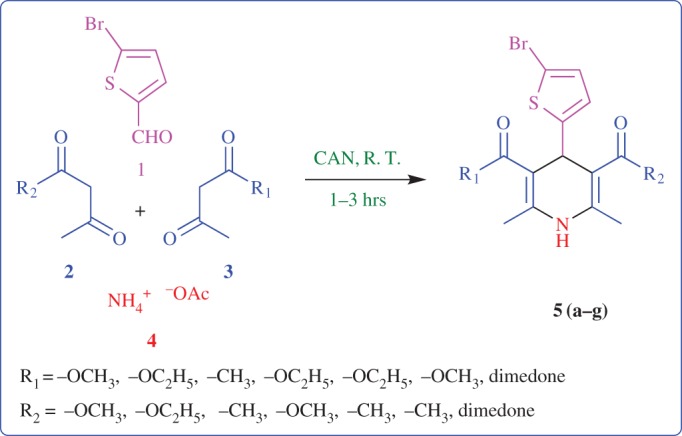
Multicomponent reaction strategies for 1,4-DHP construction.

In this study, we illustrate our recent work on the synthesis of some novel thiophene-based 1,4-DHP derivatives from different 1,3-diones, and same starting materials as 5-bromothiophen-2-carboxaldehyde and ammonium acetate under room temperature and solvent-free conditions by using ceric ammonium nitrate (CAN) as the catalyst during a short period of time; good to excellent yield is reported with a simple workup procedure, which is shown in scheme 1.

## 2. Material and methods

### 2.1. General

All reagents used in this study were purchased from commercially available sources without further purification unless specified otherwise. The synthesized compounds were characterized as anchored in ESI-MS spectra as determined by the Shimadzu GCMS-Qp 2010 spectrometer. Elemental analyses were performed in a Perkin-Elmer 2400 Series-II elemental analyser and the results were within ±0.4% of the theoretical values, unless otherwise noted. The stereochemistry and structure of all compounds were confirmed by ^1^H-NMR and ^13^C-APT spectra as recorded on Bruker Advance III (^1^H: 400 MHz) and (^13^C: 100 MHz), respectively, using CDCl_3_ as an internal standard. Splitting patterns were described as singlet (s), doublet (d), triplet (t), quartet (q) and broad (br). The values of chemical shift (δ) were given in ppm and coupling constants (J) in hertz (Hz). IR spectra were recorded on an ABB MB3000 spectrophotometer. Melting points were determined by an open capillary tube melting point apparatus and were uncorrected. The progress of reaction for compounds **(5a–f)** was monitored by silica gel 60 F254 (Merck)-coated thin-layer chromatography (TLC) plates. Reported *R*_f_ values correspond to elution with a 4 : 1 (*n*-hexane:ethyl acetate) mobile phase. Some of the compounds were separated and purified by column chromatography using an *n*-hexane:ethyl acetate (4 : 1) mobile phase. Single crystal X-ray data were collected on Rigaku SCX mini diffractometer by graphite monochromatic Mo-Kα radiation.

### 2.2. Synthesis of 1,4-dihydropyridines **(5a–5c & 5 g)**

5-Bromothiophene-2-carboxyaldehyde (1.91 g, 0.01 mol), ammonium acetate (0.77 g, 0.01 mol), different 1,3-diones (1–2.5 g, 0.01/0.02 mol) and CAN (0.28 g, 0.5 mmol) were added to a 100 ml round bottom flask. The mixture was stirred well for 1–2.5 h at room temperature, then the product was poured out and the mixture became solid. The progress of the reaction was monitored by TLC. The product was washed with water and then treated with *n*-hexane to remove impurities; after drying, the crude brown/yellow product was recrystallized using ethanol followed by charcoal treatment.

### 2.3. Synthesis of 1,4-dihydropyridines **(5d)**

5-Bromothiophene-2-carboxyaldehyde (1.91 g, 0.01 mol), ammonium acetate (0.77 g, 0.01 mol), ethylacetoacetate (1.3 ml, 0.01 mol), methylacetoacetate (1.1 ml, 0.01 mol) and CAN (0.28 g, 0.5 mmol) were added to a 100 ml round bottom flask. The mixture was stirred well for 2.5 h at room temperature; then the product was poured out and the mixture became solid. We obtained three possible products: **5a**, **5b** and **5d**. Progress of the reactions was monitored by TLC and in the case of compound **5d**, we confirmed the products by comparing spots on the TLC plate. We obtained the **5d** product in pure form, by dissolving the crude material in a (1 : 4) mixture of ethyl acetate: *n*-hexane and separating it by filtration, with a very low amount of filtrate obtained. The remaining crude material may be a mixture of the products **5a** and **5b**. In the remaining filtrate, we obtained hazy crystals of product **5d** (after 15–20 min).

### 2.4. Synthesis of 1,4-dihydropyridines **(5e & 5f)**

5-Bromothiophene-2-carboxyaldehyde (1.91 g, 0.01 mol), ammonium acetate (0.77 g, 0.01 mol), acetylacetone (1 ml, 0.01 mol), ethyl acetoacetate (1.3 ml, 0.01 mol) and CAN (0.28 g, 0.5 mmol) were added to a 100 ml round bottom flask. The mixture was stirred well for 3 h at room temperature; then the product was poured out and the mixture became solid. We obtained three possible products: **5b**, **5c** and **5e**. The progress of the reaction was monitored by TLC and in case of compound **5e**, we confirmed the products by comparing spots on the TLC plate. The *R*_f_ values for **5b**, **5c** and **5e** are 0.59, 0.19 and 0.40, respectively. Moreover, in **5f** we used methyl acetoacetate (1.1 ml, 0.01 mol) instead of ethylacetoacetate and obtained a mixture of the possible products **5a**, **5c** and **5f**. The *R*_f_ values for **5a**, **5c** and **5f** are 0.56, 0.20 and 0.46, respectively. The **5e** and **5f** compounds were separated and purified by column chromatography using an *n*-hexane: ethyl acetate (4 : 1) mobile phase.

The antimicrobial activity of the synthesized compounds **(5a–5f)** was evaluated by the broth dilution method. The synthesized compounds were tested for their antibacterial activity against Gram-negative bacteria (*E. coli* MTCC 443, *Salmonella typhi* MTCC 98) and Gram-positive bacteria (*Bacillus subtilis* MTCC 441, *Streptococcus pneumonia* MTCC 1936). Ampicillin was used as the standard against bacteria. All compounds **(5a–5f)** were screened for antifungal activity against *Candida albicans* MTCC 227, *Aspergillus niger*, MTCC 282 and *A. Clavatus* MTCC 1323 at a concentration of 500 µg ml^−1^ in DMF. Nutrient agar and potato dextrose agars were used to culture the bacteria and fungi, respectively. The plates were inculcated by the bacteria or fungi and incubated for 24 h at 37°C for bacteria and for 72 h at 27°C for fungi and then the inhibition zones of microbial growth surrounding the filter paper disc (5 mm) were measured in millimetres. Griseofulvin was used as a standard drug for fungi. Test results are given in tables 2 and 3 for antibacterial and antifungal activity, respectively.

**Table 1.** Solvent-free one-pot multicomponent reaction of 5-bromothiophene-2-carboxaldehyde (**5BT-2C**) and different 1,3-diones catalysed by CAN (ceric ammonium nitrate).

	1	2	3	4					
entry		R_1_	R_2_		time (h)	melting point (^°^C)	products **5(a–g)**	*R*_f_ value	yield (%)
1	5BT-2C	OCH_3_	OCH_3_	NH_4_OAc	1.15	180–185	**5a**	0.56	77
2	5BT-2C	OC_2_H_5_	OC_2_H_5_	NH_4_OAc	1.15	140–145	**5b**	0.59	73
3	5BT-2C	CH_3_	CH_3_	NH_4_OAc	1.15	138–142	**5c**	0.19	75
4	5BT-2C	OCH_3_	OC_2_H_5_	NH_4_OAc	2.30	118–123	**5d**	0.44	51
5	5BT-2C	CH_3_	OC_2_H_5_	NH_4_OAc	3.00	127–132	5b + 5c + **5e**	0.40	73(21 + 23 + **29**^a^)
6	5BT-2C	CH_3_	OCH_3_	NH_4_OAc	3.00	122–125	5a + 5c + **5f**	0.46	75(22 + 20 + **33**^a^)
7	5BT-2C	dimedone	dimedone	NH_4_OAc	2.30	210–213	**5 g**	0.26	35

^a^Indicates the % yield of pure compounds **5e** and **5f**.

**Table 2.** Antibacterial activity in microgram per millilitre of the synthesized compounds **(5a–5f)**. *E. coli, Escherichia coli; S. typhi, Salmonella typhi; B. subtilis, Bacillus subtilis; S. aureus, Streptococcus aureus*; A*, Ampicillin; MTCC, Microbial Type Culture collection.

compounds	*E. coli* MTCC443	*S. typhi* MTCC98	*S. aureus* MTCC96	*B. subtilis* MTCC441
**5a**	100	250	250	250
**5b**	200	500	200	100
**5c**	62.5	125	250	200
**5d**	100	200	100	62.5
**5e**	250	250	100	100
**5f**	200	100	125	62.5
A*	100	100	250	250

**Table 3.** Antifungal activity in microgram per millilitre of the synthesized compounds **5(a–f)**. *C. albicans, Candida albicans; A. niger, Aspergillus niger*.

compounds	*C. albicans*	*A. niger*
**5a**	>1000	>1000
**5b**	500	1000
**5c**	>1000	250
**5d**	>1000	500
**5e**	250	>1000
**5f**	500	500
griseofulvin	500	100

## 3. Results and discussion

We prepared a number of 1,4-DHPs via MCR. According to a literature survey for the development of synthetic procedures [18–21], mainly catalyst can play an important role in the synthesis of 1,4-DHPs [22–25]. Therefore, we selected the catalyst CAN to investigate their effect on product yield under solvent-free conditions at room temperature (table 1). Under these optimized reaction conditions, the simplicity and scope of a novel one-pot MCR protocol were explored.

The reaction of 5BT-2C **1**, ammonium acetate **4** as same starting materials and different 1,3-diones **2**,**3** was performed in the presence of CAN under solvent-free conditions to obtained desired 1,4-DHP derivatives with good to excellent yield at room temperature. Interestingly, we received the desired symmetrical products **5a**, **5b**, **5c** and **5 g** by using same 1,3-diones **2** or **3**. If we use different 1,3-diones **2**,**3**, then we get desired asymmetrical products **5d**, **5e**, **5f** including symmetrical products **5a**, **5b**, **5c** and **5f**. We obtained a **5d** product in pure form, by dissolving the crude material in a (1 : 4) mixture of ethyl acetate and n-hexane, which was filtered to separate out the undissolved crude, with a very low amount of filtrate obtained. The remaining crude may be a mixture of the products **5a, 5b** (monitored by TLC). In the remaining filtrate, we got hazy crystals of product **5d** (after 15–20 min).

However, we hypothesized properly pre-functionalized 1,3-dione **2**,**3** and 5BT-2C. In this series, 5BT-2C contains the electron-withdrawing group –Br, which increases the reactivity of aldehyde to C–C bond formation with 1,3-dione; the presence of a mild to moderate activating group in 1,3-dione increases the nucleophilicity of its methylene group to interact with 5BT-2C, as well as the susceptibility of its carbonyl groups to C–N bond formation by interaction with ammonium acetate. A series of smoothly controlled experiments clearly indicates that the 5BT-2C, ammonium acetate, 1,3-diones and CAN were all necessary substrates for this transformation, as mentioned in scheme 1.

1H-NMR data show signal in a range of 5.1–5.3δ, which confirms one proton present at the ipsolateral position on the aliphatic carbon. A singlet was observed at 1–3δ, which confirms the presence of two similar methyl groups in the meta position in the 1,4-dihydropyridine ring, with signals also for methyl and ethyl groups of esters at the desired positions. The signals of a doublet appears at 6.0–7.0δ, which confirmed the presence of aromatic protons. 13C-APT spectra show peaks in a range of 13–20δ, which confirm the presence of methyl groups. Signals at 34–35δ confirm the presence of aliphatic –CH group, whereas carbon in the pyridine ring at a double bond gave a signal at 100–110δ. Signals at 120–130δ indicate aromatic –CH groups and signals at 165–170δ confirm the presence of carbonyl groups. In the IR data, we obtained a peak near 3340 cm^−1,^ which is for N–H stretching, and a peak around 3000 cm^−1^ for tertiary –C–H stretching, whereas peaks that appear between 1700 and 1600 cm^−1^ indicate –C=O stretching. A peak that appears between 1350 and 1300 cm^−1^ indicates aromatic olefin stretching. The peak that appears near 1200 cm^−1^ provides the proof of –C–H stretching of the methyl group. The peak that appears at 510 cm^−1^ indicates –C–X (C–Br) stretching and some peaks observed between 1000 and 700 cm^−1^ confirmed the aromatic *ortho-* and *para-* di-substituents. In mass spectral analysis, we obtained peaks at the particular mass of the compound, which confirmed the present skeleton in the moiety, and different base peaks at *m/z* = 332, 318, 265, 230, 145, 57 and 45; these *m/z* values are the major base peaks in all compounds.

All the synthesized compounds **5(a–f)** were screened for Gram-positive bacteria and Gram-negative bacteria and results were compared with Ampicillin as the standard drug. Compound **5c** was found highly active, and compounds **5a** and **5d** showed equipotent activity against *E. coli* compared with the standard. Compound **5f** showed equipotent activity with *S. typhi.* The interesting finding of our work is to observe excellent antibacterial activity of the **5b, 5c, 5d, 5e** and **5f** compounds against *Staphylococcus aureus* and *Bacillus subtillus* compared with the standard drug, whereas compounds **5a** and **5c** showed equipotent activity. Similarly, the compounds **5(a–f)** were screened for antifungal activity with *C. albicans* and *A. niger* as the microorganisms; obtained results were compared with griseofulvin as the standard drug shown in table 3. It was found that compound **5e** showed higher activity than the standard. Whereas compounds **5b** and **5f** had equipotent activity with the standard, and the compounds **5a, 5c** and **5d** had lower activity.

**Figure 1.**
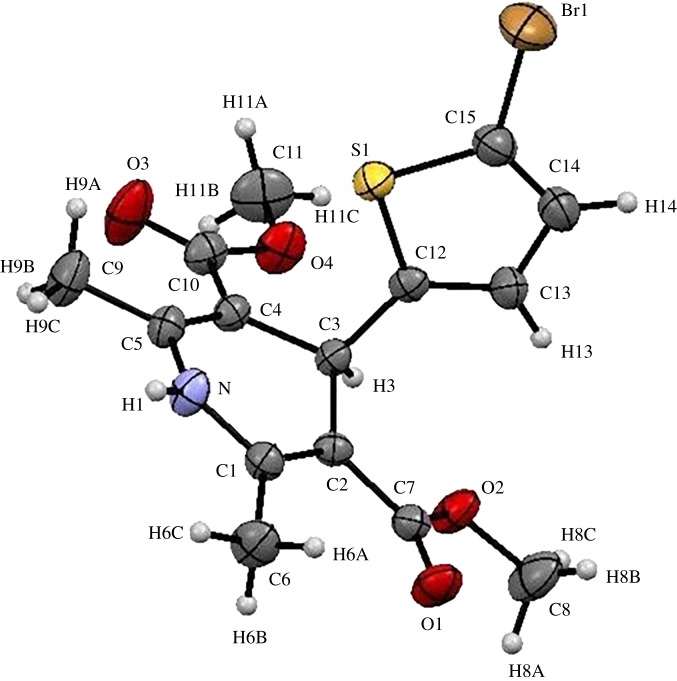
Ortep diagram for **5a**.

The cytotoxicity of all synthesized compounds was checked against breast tumour cell lines (BT-549) but no activity was found, which is illustrated in table 4. The compound **5a** was confirmed by a single crystal XRD analysis, which is shown in figures 1 and 2. The crystallographic experimental data are described in table 5. The packing arrangement of the molecules viewed down in the *a*-axis is shown along with obtained crystal structure of compound **5a**. From these series, three compounds, **5a, 5c** and **5f**, were selected for anticancer activity at National Cancer Institute (NCI), USA.

**Figurer 2.**
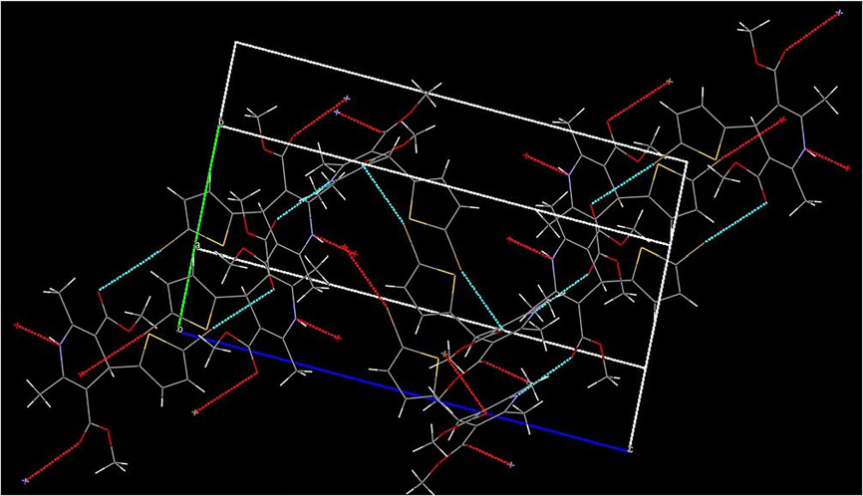
Packing arrangements of **5a** with hydrogen bonding.

**Table 4.** Cytotoxicity assay of compounds **5(a–f)** on BT-549 Cell Line in EC50 (µM).

sr. no.	compounds	EC50 (µM)
1	**5a**	42.0
2	**5b**	52.5
3	**5c**	>100
4	**5d**	45.5
5	**5e**	81.8
6	**5f**	77.7
7	doxorubicin	0.04

**Table 5.** Crystal and experimental data of compound **5a**.

CCDC No	1471153
crystal description	colourless block crystal
crystal size	0.480 × 0.390 × 0.310 mm
empirical formula	C_15_H_16_BrNO_4_S
formula weight	386.26
radiation, wavelength	MoKα(*λ* = 0.71075 Å) graphite monochromatic
unit cell dimensions	*a* = 8.0516(9) Å, *b* = 10.072(1) Å, *c* = 20.119(2) Å, *β* = 97.803(4)°
crystal system	monoclinic
lattice type	primitive
space ground	P2_1_/c (#14)
unit cell volume	*V* = 1616.4(3) Å^3^
calculated density	1.587 g cm^−3^
no. of molecules per unit cell, Z	4
µ (MoKα)	26.958 cm^−1^
*F*(000)	784.00
refinement of unit cell	full-matrix least-squares on *F*^2^
reflection/parameter ratio	18.61
residuals: R1 (*I* > 2.00 *σ* (*I*))	0.0437
*R*_int_	0.0360
least-squares weights	*w* = 1/[*σ*^2^(Fo^2^) + (0.1000 ^.^ *P*)^2^ + 0.0000 ^. ^*P*], where *P* = (Max(Fo^2^,0) + 2Fc^2^)/3
(*Δ*/*σ*)_max_ in the final cycle	15.000
goodness of fit indicator	1.125
maximum peak in final diff. map	0.75 e^−^ Å^−3^
minimum peak in final diff. map	−0.53 e^−^ Å^−3^
2*θ*_max_ cut-off	55.0^°^
function minimized	Σ *w* (Fo^2^ − Fc^2^)^2^
*ω* oscillation range	−120.0 − 60.0°
exposure rate	10.0 s per degree
detector swing angle	−30.80°
diffractometer	SCX mini

## 4. Conclusion

In brief, we have confirmed a well-organized solvent-free green procedure for the multicomponent synthesis of dihydropyridine derivatives using the catalyst CAN. The result highlighted the solvent-free procedure to be more efficient than the conventional method. The advantages of the Hantzsch pyridine synthesis are shorter reaction times, simplicity of the reaction, good product yield and easy workup procedures with regard to the build-up to the reaction, which is economical and easy, with CAN being a powerful catalyst for the many organic syntheses. The interesting finding of our work is that we obtained excellent antibacterial activity with compounds such as **5b**, **5c**, **5d**, **5e** and **5f**, which were found highly active against *S. aureus* and *B. subtillus* compared with the standard drug Ampicillin, whereas compounds **5a** and **5c** showed equipotent activity. As far as antifungal activity is concerned, only compound **5e** showed higher activity than the standard griseofulvin drug, and compounds **5b** and **5f** had equipotent activity. The cytotoxicity of all the compounds has been assessed against breast tumour cell lines (BT-549), but no activity was found.

## Supplementary Material

Electronic supplementary material

## Data accessibility

We include all the experimental data including spectral characterizations in the electronic supplementary material. ESI and crystallographic details of **5a** compound are available on http://dx.doi.org/10.5061/dryad.66gd7 [26], and other figures and electronic supplementary materials are available on https://doi.org/10.6084/m9.figshare.4990370.
