# Supplementary material for: Green approach for synthesis of bioactive Hantzsch 1,4-dihydropyridine derivatives based on thiophene moiety via multicomponent reaction
Source: R Soc Open Sci. 2017 Jun 14;4(6):170006. doi: 10.1098/rsos.170006 (PMC5493906; doi:10.1098/rsos.170006)
Supplement: Electronic supplementary material [file rsos170006supp1.docx]

**Supplementary information**

**Green approach for synthesis of bioactive Hantzsch 1, 4-dihydropyridine derivatives based on thiophene moiety via multicomponent reaction**

**Sharma^1^ MG, Rajani^2^ DP, Patel HM^1*^**

*Department of Chemistry, Sardar Patel University, Vallabh Vidyanagar-388120, Gujarat, India, Email:* [*hm_patel@spuvvn.edu*](mailto:hm_patel@spuvvn.edu)

**Table of contents:**

**Data for Hantzsch pyridine synthesis 1-4**

**^1^H NMR, ^13^C APT, selected IR and Mass spectra 5-16**

**Single Crystal Study for A2 16**

**Data for Hantzsch pyridine synthesis**

**5a**

**Dimethyl4-(5-bromothiophene-2-yl)1,4-dihydro-2,6-dimethylpyridine-3,5-carboxylate (5a)** Prepared from 5-bromothiophene-2-carbaldehyde(1.91 g, 0.01mol), methyl acetoacetate (2.32 g, 0.02 mol ) and ammoniumacetate (0.77 g, 0.01 mol) and CAN {0.28 g, 0.5 mol (5%)}; produce in pure form; **Yield:** 2.98 g, 77 **%**; light brown solid; **mp:** 180-185^O^C; **R_f_ value:** (n-hexane: ethyl acetate, 1:1) 0.56; **^1^H-NMR** (CDCl_3 ,_ 400 MH_Z_); δ = 2.27 (s, 6H) , 3.65 (s, 6H), 5.17 (s, 1H), 5.88 (s, 1H), 6.46 (dd, *J*= 3.6, 0.8 Hz, 1H), 6.72 (d, *J*= 4 Hz, 1H) ppm; **^13^C -APT** (CDCl_3_, 100MH_Z_); δ = 14.3 (CH_3_), 19.4 (CH_3_), 34.9 (CH), 60.0 (CH_2_), 102.9 (C), 109.5 (C), 123.3 (CH), 129.1 (CH), 144.8 (C), 153.0 (C), 167.1 (C=0) ppm; **IR** (neat) ν= 3348, 3340, 2947, 1662, 1643, 1481, 1427, 1381, 1304, 1095, 1049, 1018, 748, 579; **Mass**: m/z = 385, 332, 318, 304, 230, 216, 145, 45; **Elemental analysis**: (%) calculated for C_15_H_16_BrNO_4_S: C 46.64, H 4.18, N 3.63; Found: C 46.48, H 4.04, N 3.27

5b

**Diethyl4-(5-bromothiophene-2-yl)1,4-dihydro-2,6-dimethylpyridine-3,5-carboxylate (5b)** Prepared from 5-bromothiophene-2-carbaldehyde(1.91 g, 0.01mol), ethyl acetoacetate (2.64 g, 0.02 mol ) and ammonium acetate (0.77 g, 0.01 mol); CAN {0.28 g, 0.5 mol (5%)}; product in pure form ; **Yield:** 2.98 g, 3.02 g, 73 **%**; light brown solid; **mp:** 140-145^O^C; **R_f_ value:** (n-hexane: ethyl acetate, 1:1) 0.59;  **^1^H-NMR** (CDCl_3 ,_ 400 MH_Z_) δ = 1.27-1.31 (t, 6H) , 2.34 (s, 6H), 4.15-4.23 (m, 4H), 5.26 (s, 1H), 6.05 (s,1H), 6.56 (d, *J*= 3.6 Hz, 1H), 6.80 (d, *J*= 4 Hz, 1H) ppm; **^13^C -APT** (CDCl_3_, 100MH_Z_) δ = 14.3 (CH_3_), 19.4 (CH_3_), 34.9 (CH), 60.0 (CH_2_), 102.9 (C), 109.5 (C) , 123.3 (CH), 129.1 (CH), 144.8 (C), 153.0 (C), 167.1 (C=0) ppm; **IR** (neat) ν=3340, 1690, 1643, 1489, 1443 , 1373, 1296, 1265, 1211, 1119, 1095, 1018; **Mass**: m/z = 413, 368, 332, 304, 251, 232, 206, 145, 57, 45; **Elemental analysis :** (%) Calculated for C_17_H_20_BrNO_4_S: C 49.28, H 4.87, N 3.38; Found: C 49.06, H 4.72, N 3.24.

**5c**

**4-(5-bromothiophene-2-yl)-3,5-diacetyl-1,4-dihydro-2,6-dimethylpyridine(5c)** Prepared from 5-bromothiophene-2-carbaldehyde(1.91 g, 0.01 mol), acetylacetone (2.04 g, 0.02 mol ) and ammonium acetate (0.77 g, 0.01 mol); CAN {0.28 g, 0.5 mol (5%)}; product in pure form ; **Yield:** 2.64 g, 75 **%**; yellowish solid; **mp:** 138-142^O^C; **R_f_ value:** (n-hexane: ethyl acetate, 1:1) 0.19; **^1^H-NMR** (CDCl_3 ,_ 400 MH_Z_) δ = 2.24 (s, 6H), 2.27 (s, 6H), 5.23 (s, 1H), 6.45 (dd, *J*=3.6, 0.8 Hz, 1H), 6.50 (s,1H), 6.72 (d, *J*=3.6 Hz, 1H) ppm; **^13^C -APT** (CDCl_3_, 100 MH_Z_) δ = 20.3, 30.0, 35.5, 110.4, 112.9, 123.7, 129.3, 144.3, 151.7, 197.0 ppm; **IR** (neat) ν=3310, 3055, 1674, 1589, 1466, 1427, 1366, 1327, 1210, 1157, 1119, 1028, 957, 795, 517; **Elemental analysis:** (%) Calculated for C_15_H_16_BrNO_2_S: C 50.86, H 4.55, N 3.95; Found: C 50.84, H 4.52, N 3.92.


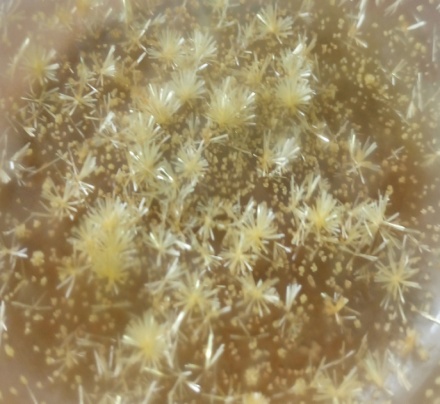


5d

3-**ethyl,5-methyl,4-(5-bromothiophene-2-yl)1,4-dihydro-2,6-dimethylpyridine-3,5-carboxylate(5d)** Prepared from 5-bromothiophene-2-carbaldehyde (1.91 g, 0.01 mol), ethylacetoacetate (1.32 g, 0.01 mol) methylacetoacetate (1.16 g, 0.01 mol) and ammonium acetate (0.77 g, 0.01 mol); CAN {0.28 g, 0.5 mol (5%)}; mixture of products **5a**,**5b** and **5d**, dissolved the mixture in (8:2) N-hexane : ethyl acetate solution and then filter it, light yellow colored crystals of **5d** was generated in filtrate.; **Yield:** 2.02 g, 51 **%**; yellow solid crystals; **mp:** 118-123^O^C; **R_f_ value:** (n-hexane: ethyl acetate, 1:1) 0.44; **^1^H-NMR** (CDCl_3 ,_ 400 MH_Z_) δ = 1.20 (t , 3H) , 2.27 (s , 6H) , 3.65 (s, 3H) , 4.13 ( q, 2H) , 5.17 (s, 1H) , 5.86 (d, 1H) 6.47 (dd, *J*=2.8, 0.4 Hz, 1H) , 6.72 (dd, *J*=4, 1.2 Hz, 1H) ppm; **^13^C -APT** (CDCl_3_, 100MH_Z_) δ =14.35 , 19.53 , 34.86 , 51.24 , 60.1 , 77.0 , 109.6 , 123.3, 129.2, 144.8 , 145.8 , 167.1 ppm; **IR** (neat) ν=3340, 1697, 1643, 1489, 1435, 1373, 1342, 1296, 1265, 1211, 1157, 1095, 1026, 748, 509; **Mass**: m/z =399, 383, 332, 318, 304, 290, 258, 230, 192, 145, 133, 91, 45.; **Elemental analysis :** (%) Calculated for C_16_H_18_BrNO_4_S: C 48.01, H 4.53, N 3.50; Found: C 47.89, H 4.32, N 3.38.

5e

**Ethyl-5-acetyl4-(5-bromothiophene-2-yl)1,4-dihydro-2,6-dimethylpyridine-3-carboxylate(5e)** Prepared from 5-bromothiophene-2-carbaldehyde (1.91 g, 0.01 mol), ethylacetoacetate (1.32 g, 0.01 mol) acetyl acetone(1.02 g, 0.01 mol) and ammonium acetate (0.77 g, 0.01 mol); CAN {0.28 g, 0.5 mol (5%)}; mixture of products **5b**, **5c** and **5e**, (yield: 3.55 g, 73%) separated and purified by column chromatography, **Yield:** 1.42 g, 29 **%**; yellowish solid; **mp:** 137-139 ^O^C; **R_f_ value:** (n-hexane: ethyl acetate, 1:1) 0.40;  **^1^H-NMR** (CDCl_3 ,_ 400 MH_Z_) δ =1.19-1.21 (t, 3H), 2.27 (s, 6H), 3.65 (s, 3H), 4.10-4.13 (q, 2H), 5.17 (s, 1H), 5.88 (s, 1H), 6.46 (dd, *J=*3.6 0.8 Hz, 1H), 6.72 (d, *J=*4.1 Hz, 1H) ppm; **Mass**: m/z = 383, 332, 302, 229, 229, 214, 149, 111, 85, 57, 55.; **Elemental analysis :** (%) Calculated for C_16_H_18_BrNO_3_S: C 50.01, H 4.72, N 3.64; Found: C 49.89, H 4.67, N 3.58.

5f

**Methyl-5-acetyl4-(5-bromothiophene-2-yl)1,4-dihydro-2,6-dimethylpyridine-3-carboxylate(5f)** Prepared from 5-bromothiophene-2-carbaldehyde (1.91 g, 0.01 mol), methylacetoacetate (1.16 g, 0.01 mol) acetylacetone(1.02 g, 0.01 mol) and ammoniumacetate (0.77 g, 0.01 mol); CAN {0.28 g, 0.5 mol (5%)} ; mixture of products **5a**, **5c** and **5f** (yield: 3.70 g, 75 %) separated and purified by column chromatography; **Yield:** 1.20 g, 33 **%**; yellowish solid; **mp:** 135-139 ^O^C; **R_f_ value:** (n-hexane: ethyl acetate, 1:1) 0.46;  **^1^H-NMR** (CDCl_3 ,_ 400 MH_Z_) δ =2.24 (s, 6H), 2.27 (s, 3H), 3.62 (s, 3H), 5.15 (s, 1H), 5.87 (s, 1H), 6.46 (dd, *J=* 4.4, 0.8 Hz, 1H), 6.72 (d, *J=* 4.8 Hz, 1H) ppm ; **Elemental analysis :** (%) Calculated for C_15_H_16_BrNO_3_S: C 48.66, H 4.36, N 3.78; Found: C 48.26, H 4.34, N 3.67.

5g

**9-(5-bromothiophene-2-yl)3,4,6,7-tetrahydro-3,3,6,6-tetramethylacridine 1,8(2H,5H, 9H,10H)dione(5g)** Prepared from 5-bromothiophene-2-carbaldehyde (1.91 g, 0.01 mol), dimedone (2.80 g, 0.02 mol) and ammonium acetate (0.77 g, 0.01 mol); CAN {0.28 g, 0.5 mol (5%)}; product formed with some intermediates/impurities, separated by column chromatography ; **Yield:**  1.50 g, 35 %; yellowish solid; **mp:** 170-174 ^O^C; **R_f_ value:** (n-hexane: ethyl acetate, 1:1) 0.26;  **^1^H-NMR** (CDCl_3 ,_ 400 MH_Z_) δ =0.97 (s, 6H), 1.02 (s, 6H), 2.20 (s, 4H), 2.24 (s, 4H), 5.26 (s, 1H), 6.52 (d, *J=*3.6 Hz, 1H), 6.66 (d, *J=*3.6 Hz, 1H), 7.19 (s, 1H) ppm; **^13^C -APT** (CDCl_3_, 100MH_Z_) δ = 27.2, 29.5, 32.6, 40.6, 50.8, 109.3, 111.7, 124.0, 129.5, 150.3, 151.9, 196.1 ppm.; **Elemental analysis :** (%) Calculated for C_21_H_24_BrNO_2_S: C 58.07, H 5.57, N 3.22; Found: C 58.08, H 5.50, N 3.20.

**^1^H NMR, ^13^C APT, selected IR and Mass spectra Compound 5a**
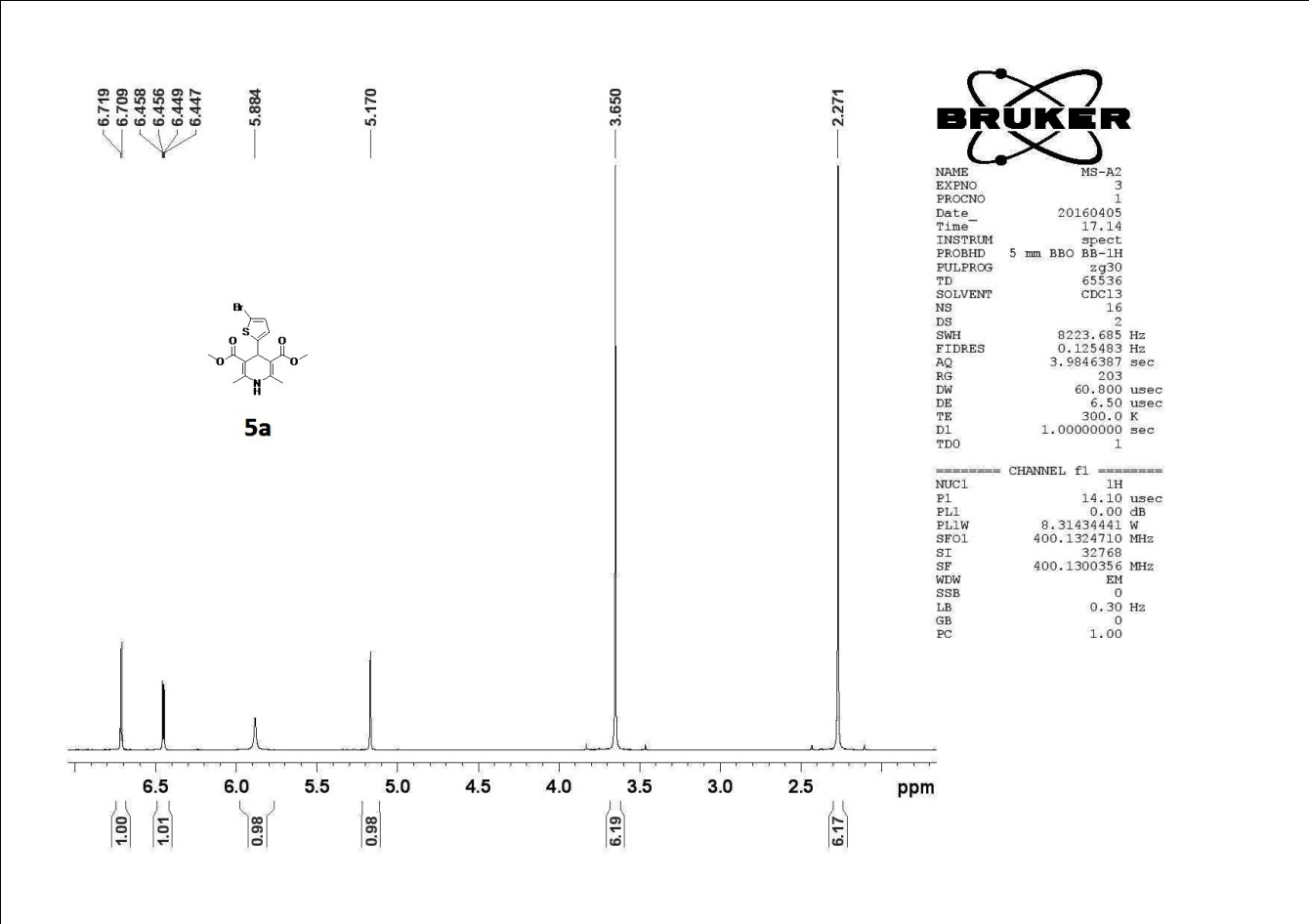

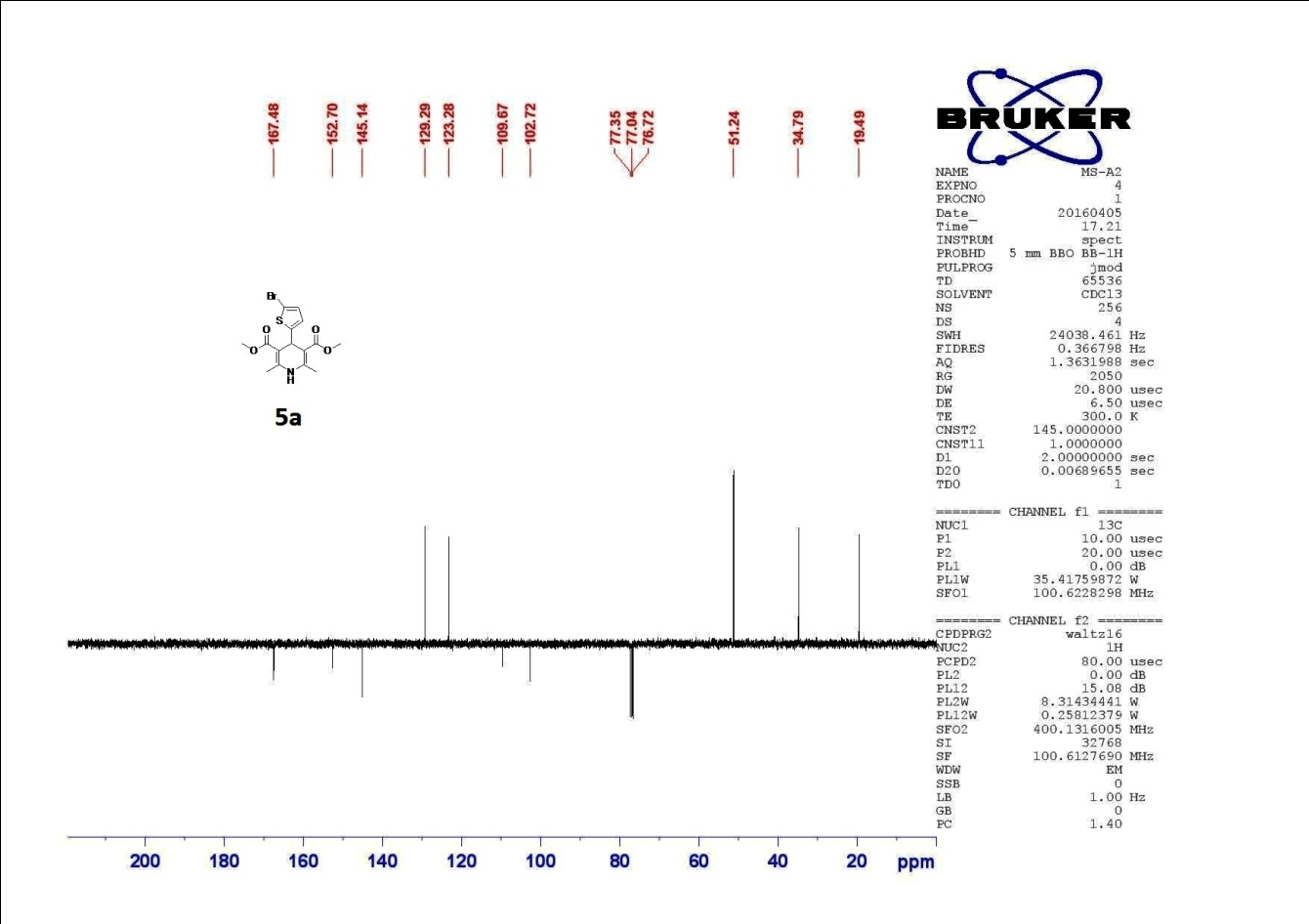


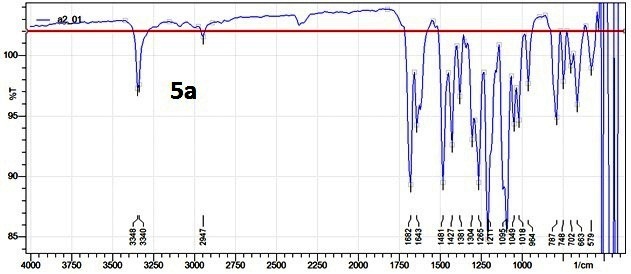


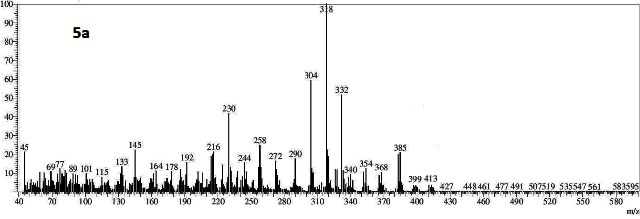


**Compound 5b**
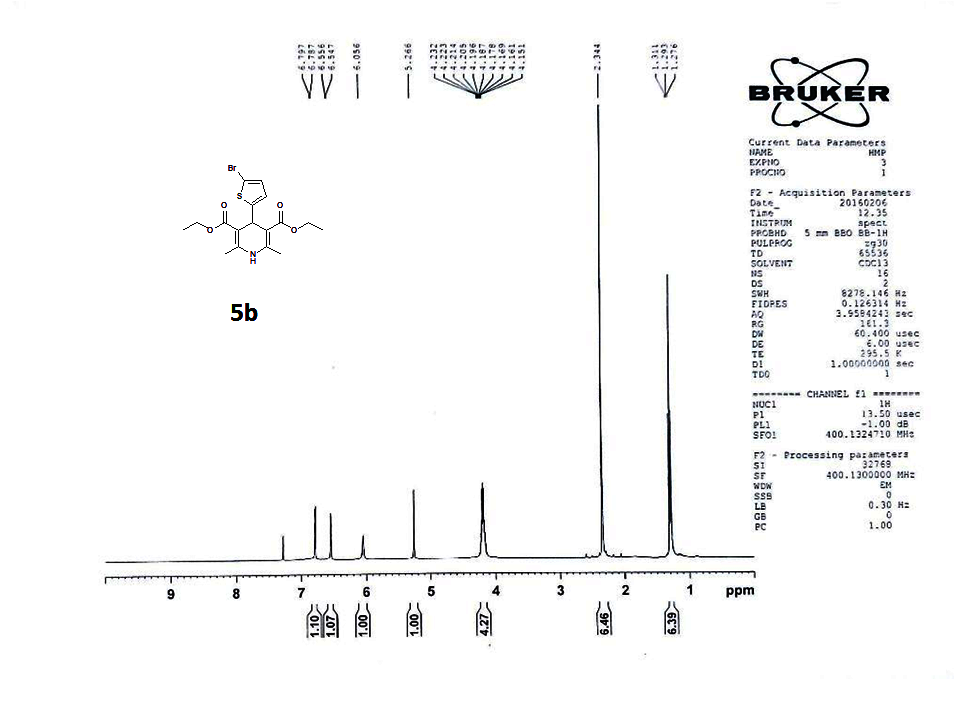


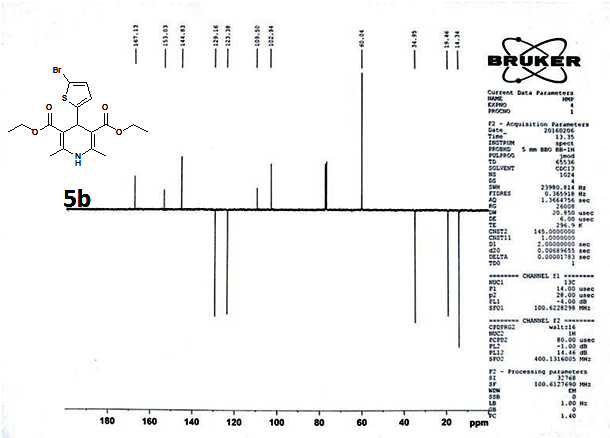


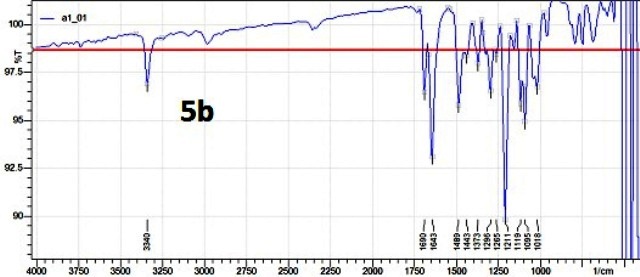


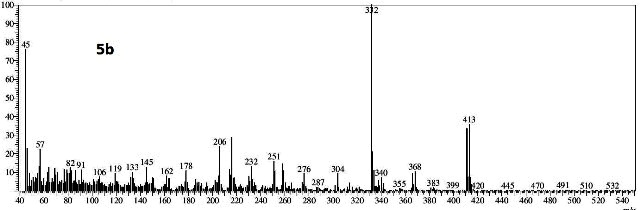


**Compound 5c**
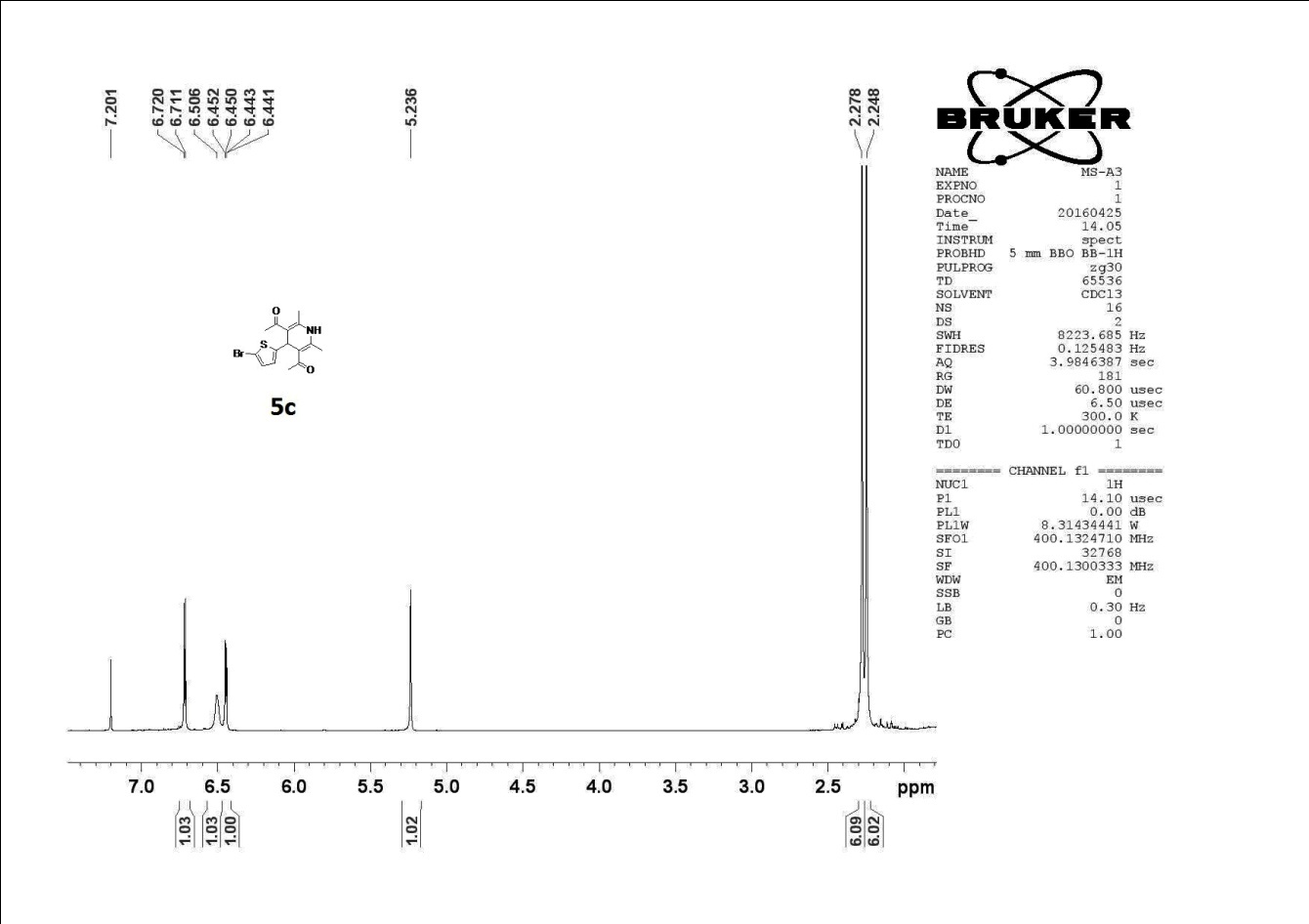


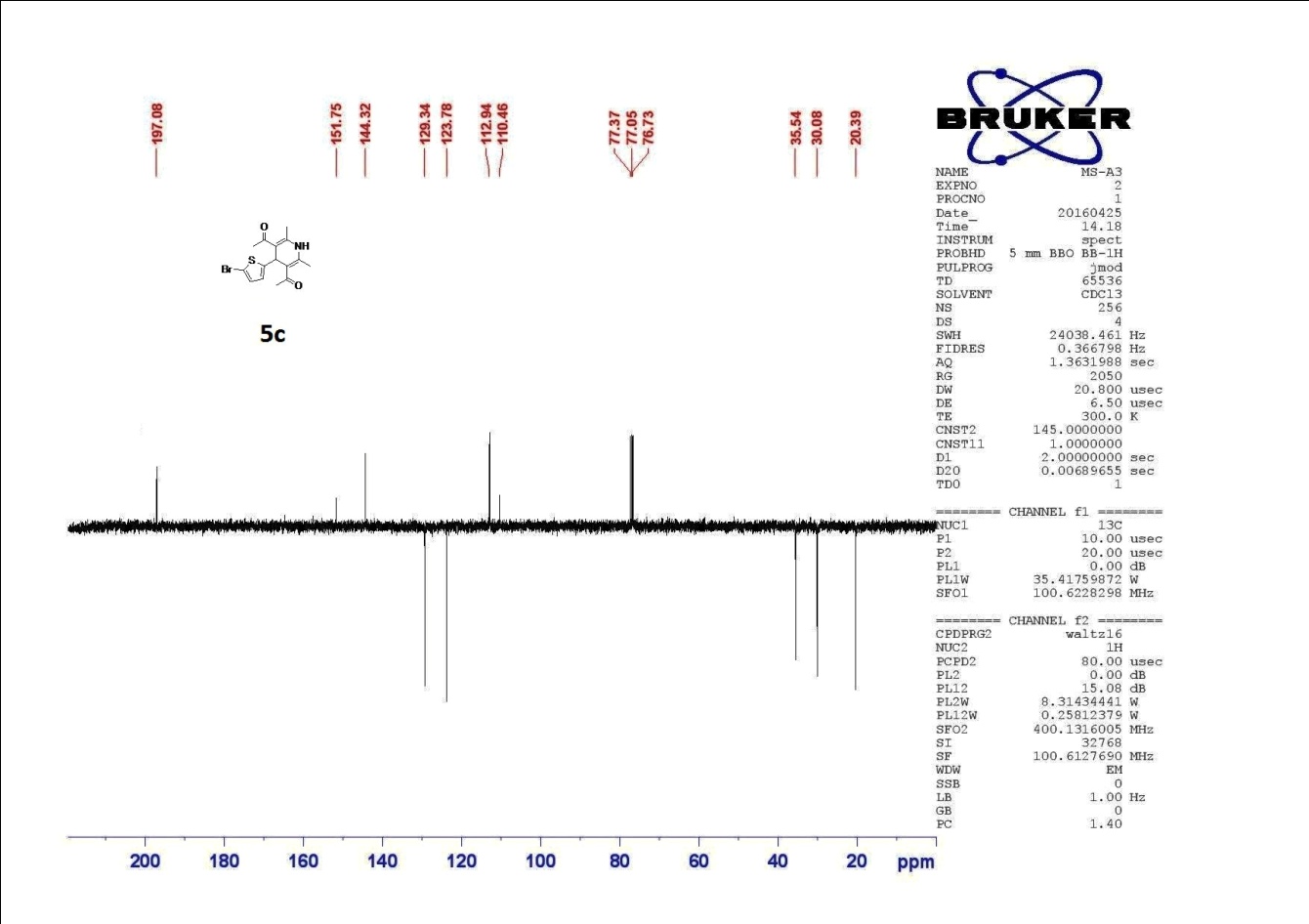


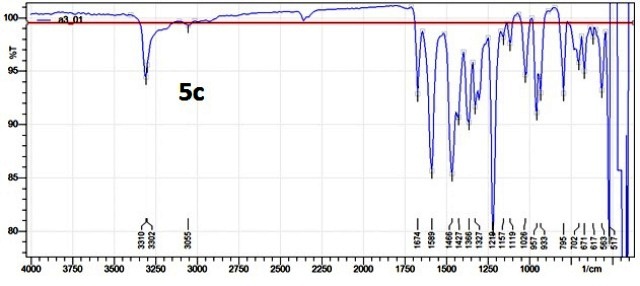


**Compound 5d**


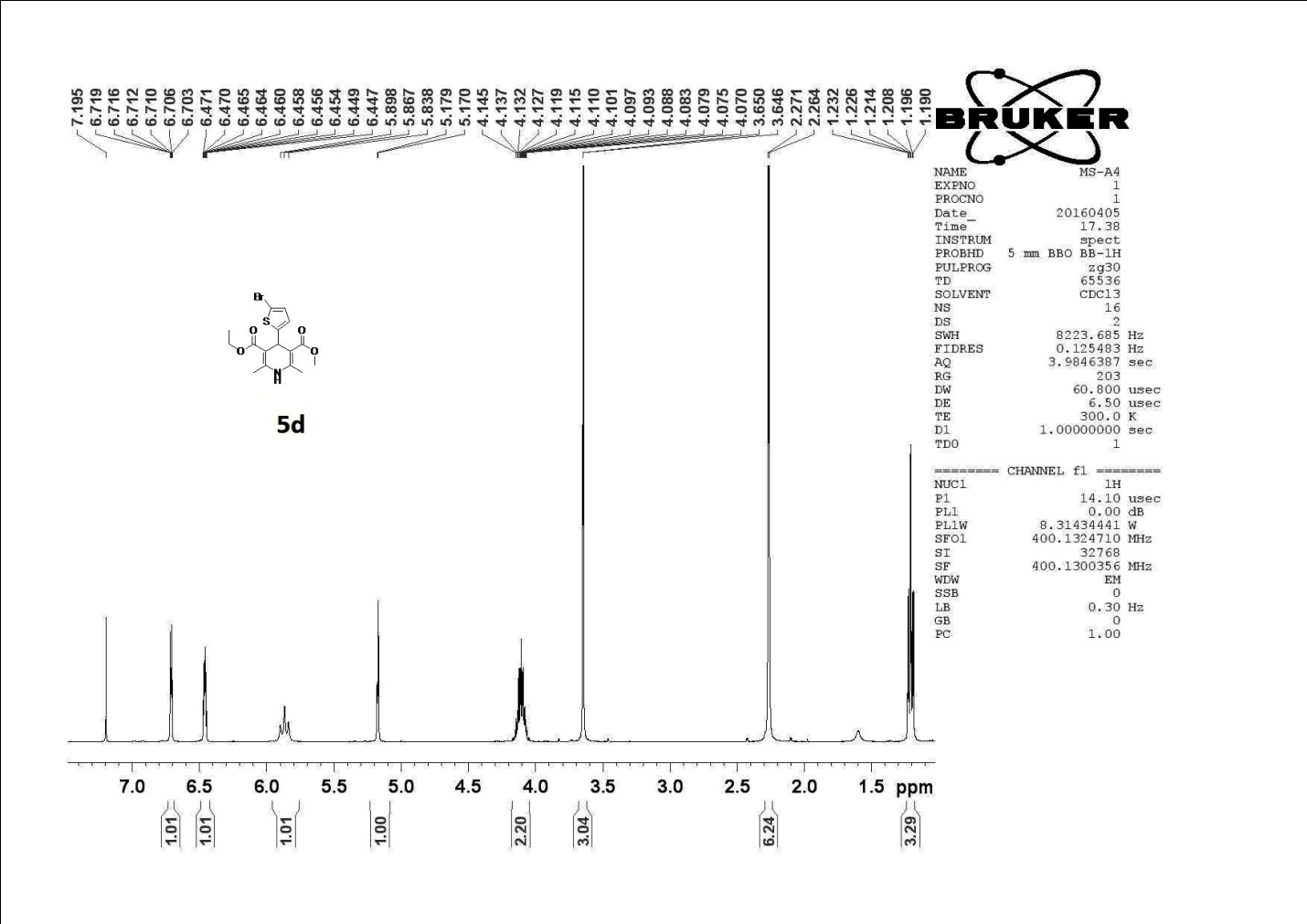


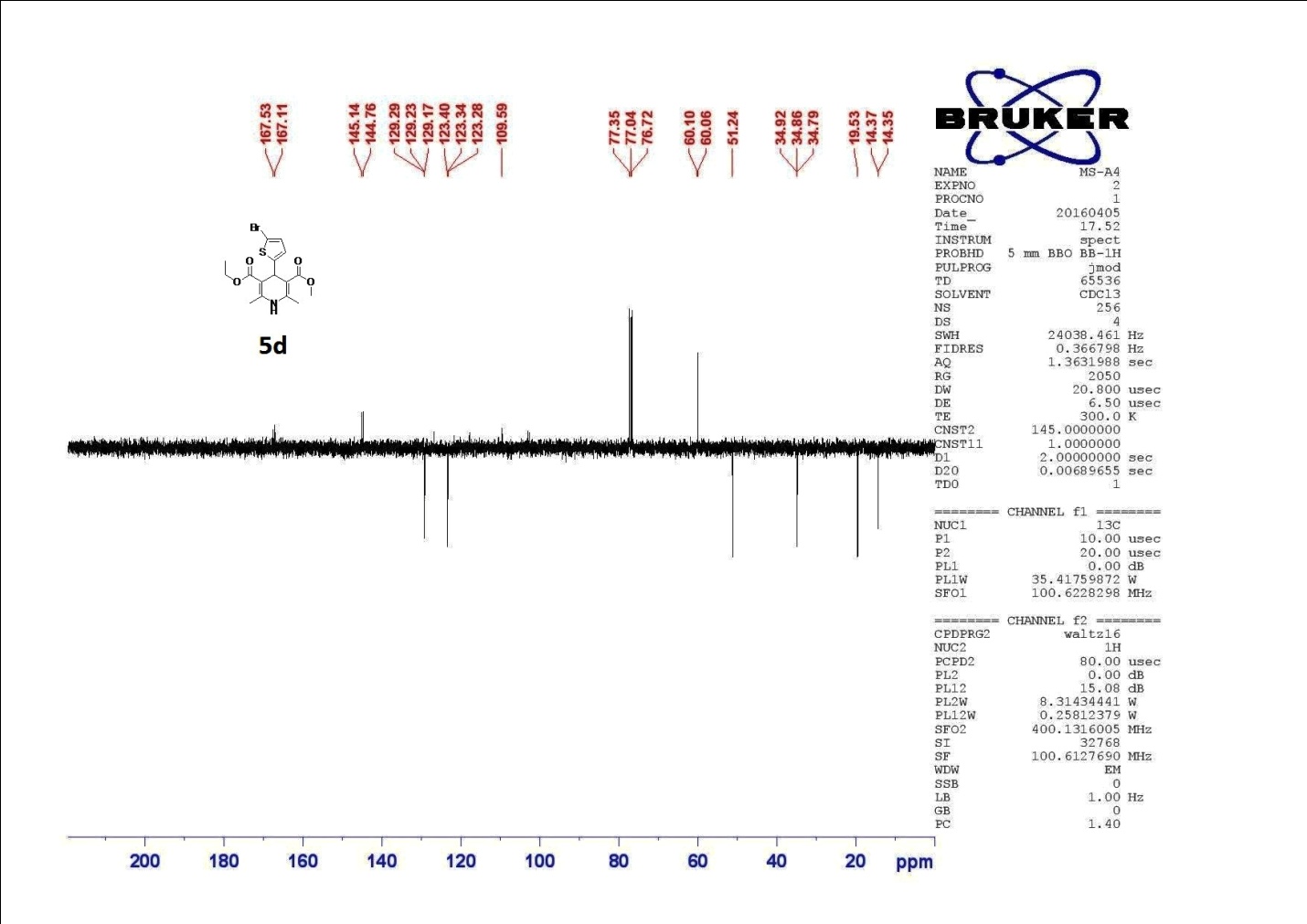


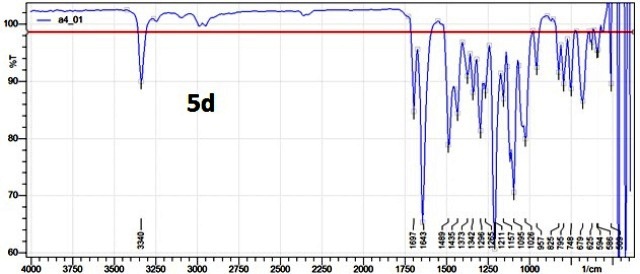


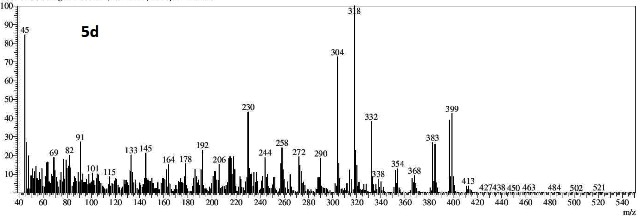


**Compound 5e**


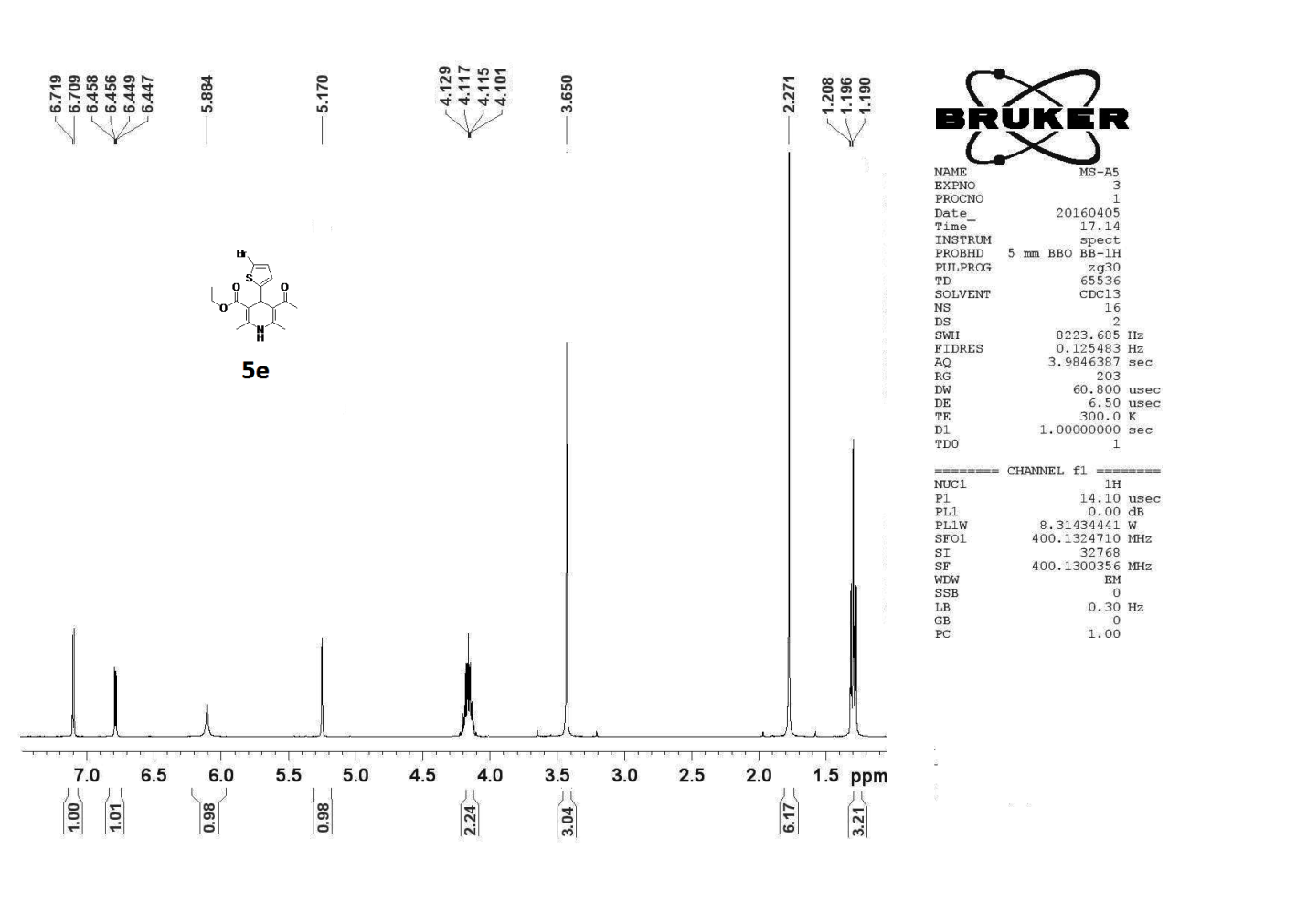


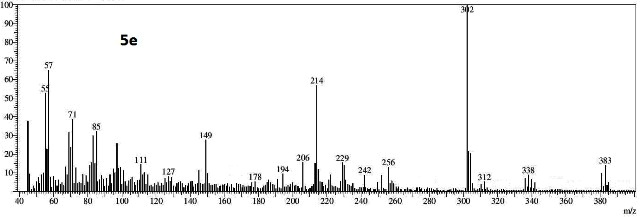


**Compound 5f**


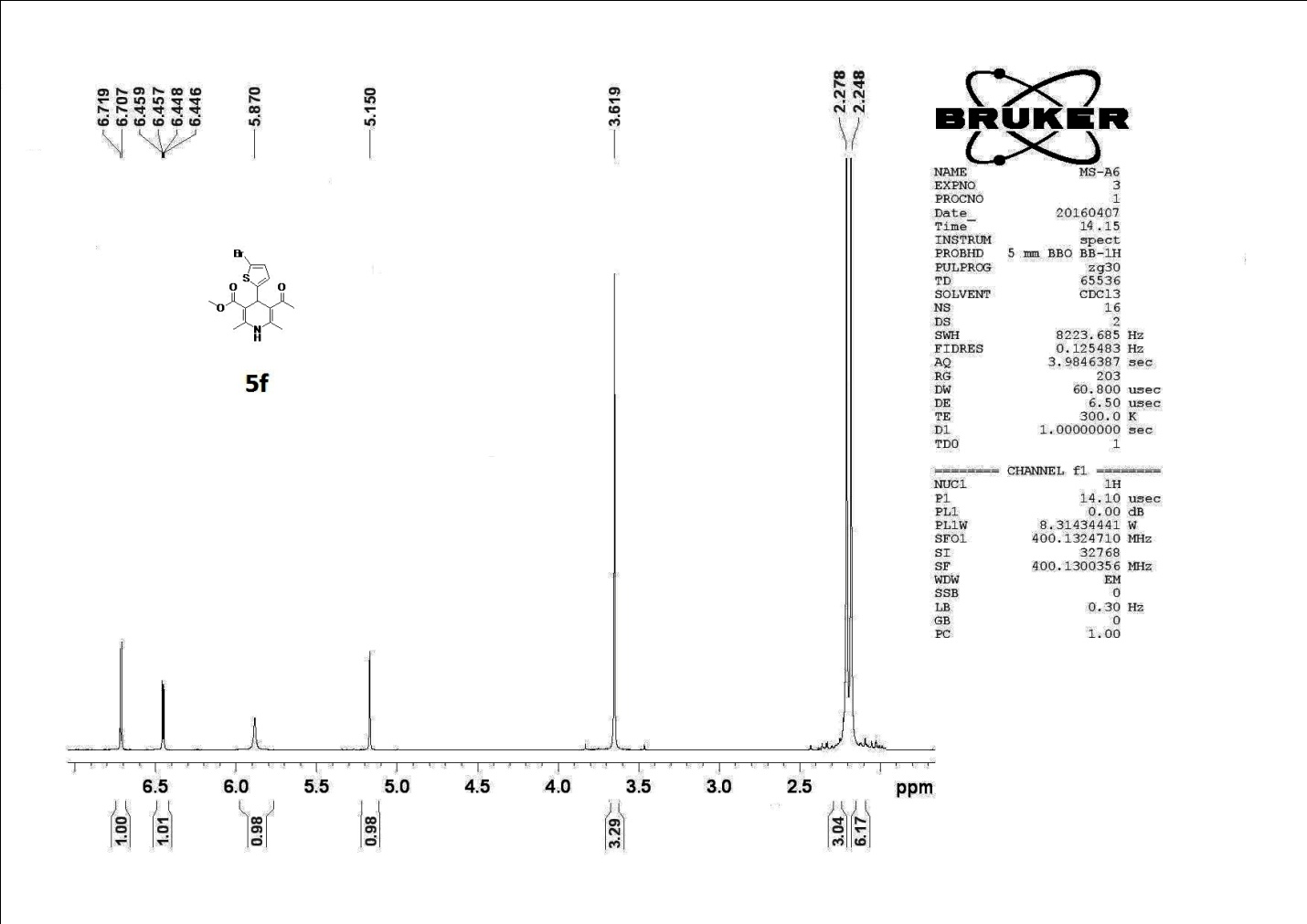


**Compound 5g**


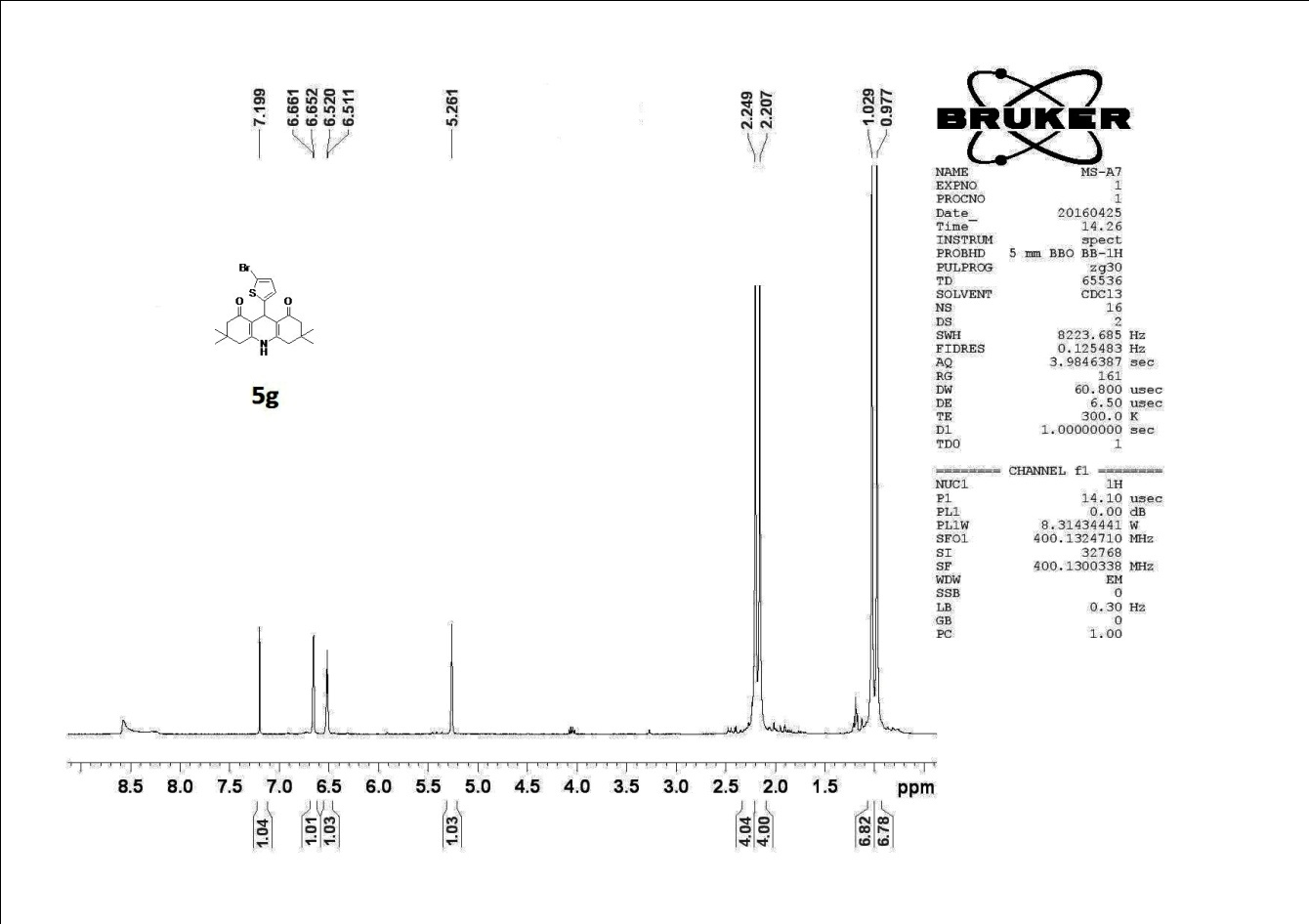


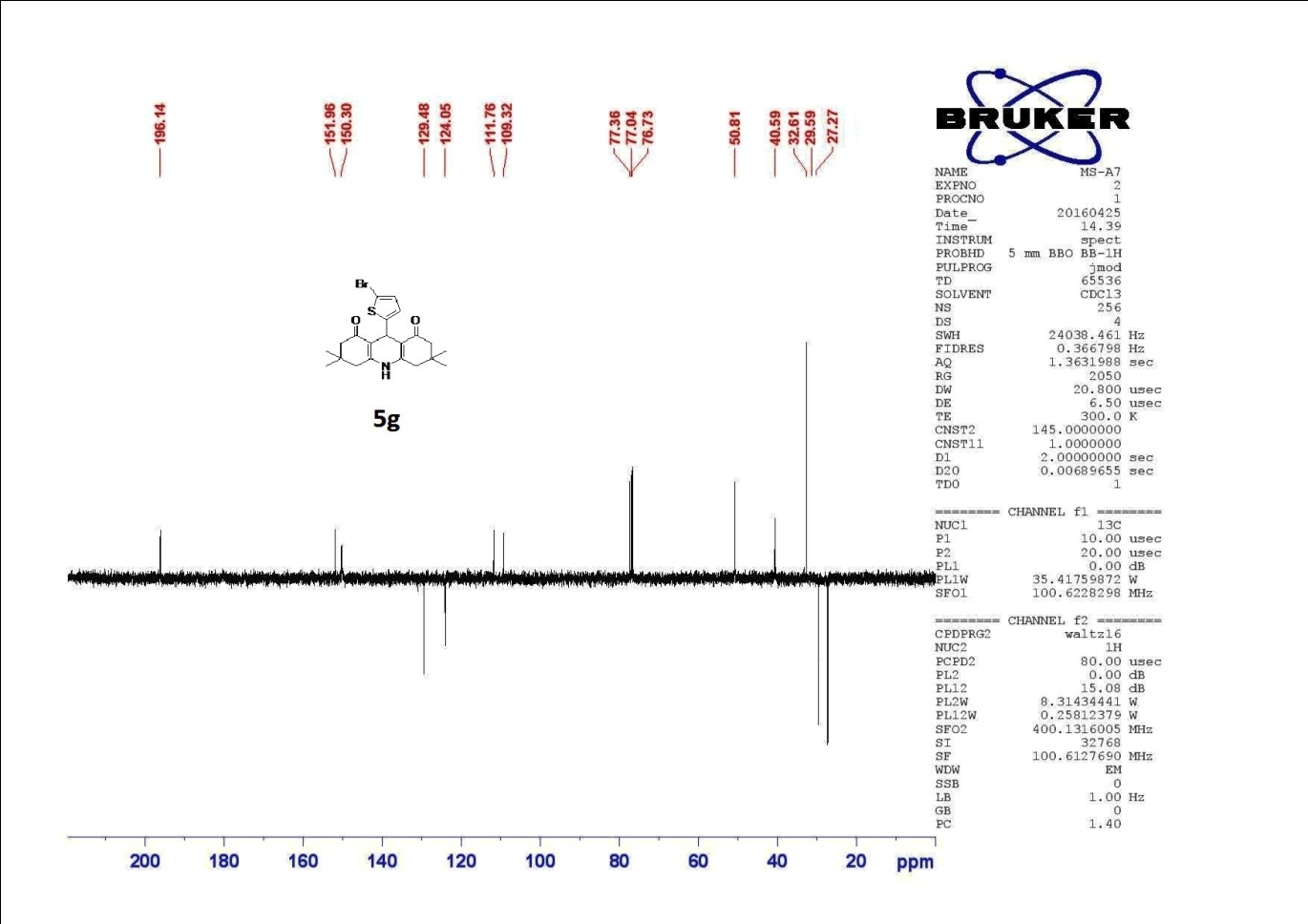


**Single Crystal Study for compound 5a.
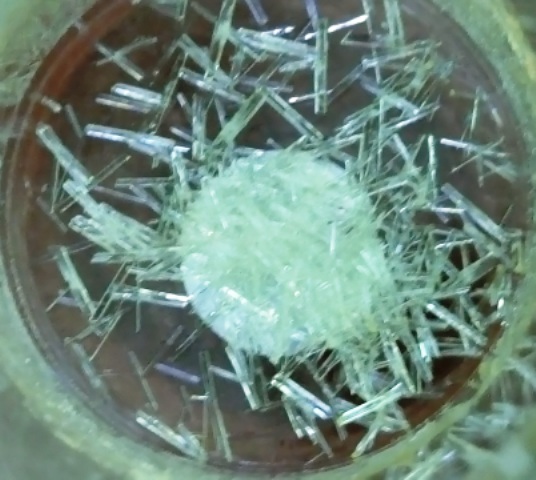
**

Crystals were grown from C_2_H_5_OH followed by charcoal treatment. A colorless block crystal of C15H16BrNO4S having approximate dimensions of 0.480 x 0.390 x 0.310 mm was mounted on a glass fiber. All measurements were made on a Rigaku SCX mini diffractometer using graphite monochromatic Mo-Kα radiation. The crystal-to-detector distance was 52.00 mm. Cell constants and an orientation matrix for data collection corresponded to a primitive monoclinic cell with dimensions:

a = 8.0516(9) Å, b= 10.072(1) Å, c = 20.119(2) Å, β = 97.803(4)o, V = 1616.4(3) Å3

For Z = 4 and F.W. = 386.26, the calculated density is 1.587 g/cm3. The reflection conditions of: h0l: l = 2n, 0k0: k = 2n

Uniquely determine the space group to be: P21/c (#14)

The data were collected at a temperature of 20 + 1oC to a maximum 2θmax cutoff value of 55.0o. A total of 540 oscillation images were collected. A sweep of data was done using  oscillations from -120.0 to 60.0o in 1.0o steps. The exposure rate was 10.0 [sec./o]. The detector swing angle was -30.80o. A second sweep was performed using  oscillations from -120.0 to 60.0o in 1.0o steps. The exposure rate was 10.0 [sec./o]. The detector swing angle was -30.80o. Another sweep was performed using  oscillations from -120.0 to 60.0o in 1.0o steps. The exposure rate was 10.0 [sec./o]. The detector swing angle was -30.80o. The crystal-to-detector distance was 52.00 mm. Readout was performed in the 0.146 mm pixel mode.
